# Trends in the incidence of brain cancer and the use of mobile phones: analysis of the Spanish Network of Cancer Registries (REDECAN)

**DOI:** 10.1007/s12094-025-03932-y

**Published:** 2025-05-14

**Authors:** Jaume Galceran, Alberto Ameijide, Adela Cañete, Rafael Peris-Bonet, Arantza López de Munain, Amaia Aizpurúa, Marta de la Cruz, Arantza Sanvisens, María José Sánchez, Isabel Palacios, Paula Franch, Antonia Sánchez, Marcela Guevara, Marià Carulla, Pilar Gutiérrez, Isabel Sáez, Marta Rodríguez, Araceli Alemán, Consol Sabater

**Affiliations:** 1 Tarragona Cancer Registry, Cancer Epidemiology and Prevention Service, Sant Joan de Reus University Hospital, Reus, Tarragona Spain; 2 Pere Virgili Health Research Institute (IISPV), Reus, Tarragona Spain; 3 Paediatric Oncohematology Unit, Hospital La Fe, Valencia, Spain; 4 Spanish Registry of Childhood Tumours (RETI-SEHOP), Universitat de Valencia, Valencia, Spain; 5 Department of Paediatrics, University of Valencia, Valencia, Spain; 6 Basque Country Cancer Registry , Department of Health, Basque Government, Vitoria-Gasteiz, Spain; 7 Epidemiology Unit and Girona Cancer Registry, Oncology Coordination Plan, Catalan Institute of Oncology, Girona, Spain; 8 Girona Biomedical Research Institute Dr. Josep Trueta (IDIBGI), Girona, Spain; 9 Granada Cancer Registry, Andalusian School of Public Health (EASP), Granada, Spain; 10 Instituto de Investigación Biosanitaria Ibs.GRANADA, Granada, Spain; 11 Department of Preventive Medicine and Public Health, University of Granada, Granada, Spain; 12 Consortium for Biomedical Research in Epidemiology and Public Health (CIBERESP), Madrid, Spain; 13 La Rioja Cancer Registry, Epidemiology and Health Prevention Service , Logroño, Spain; 14 Mallorca Cancer Registry, Public Health and Participation Department, Palma, Spain; 15 Health Research Institute of the Balearic Islands (IdISBa), Palma, Spain; 16 Murcia Cancer Registry , Department of Epidemiology, Regional Health Authority, Instituto Murciano de Investigación Biosanitaria (IMIB)-Arrixaca, Murcia University, Murcia, Spain; 17 Navarra Cancer Registry, Navarra Public Health Institute, Pamplona, Spain; 18 Epidemiology and Public Health Area, Navarra Institute for Health Research (IdiSNA), Pamplona, Spain; 19 Castilla y León Cancer Registry, Public Health Directorate, Castilla y León Government, Valladolid, Spain; 20 Registry of Childhood and Adolescent Tumours of the Valencian Community, Generalitat Valenciana, Valencia, Spain; 21 Asturias Cancer Registry, Public Health Directorate, Asturias, Spain; 22 Canary Islands Cancer Registry, Public Health Directorate, Canary Islands Government, Tenerife, Spain; 23 Castellón Cancer Registry, Directorate General of Public Health and Addictions, Valencian Government, Castellón, Spain

**Keywords:** Brain tumours, Incidence trends, Mobile phones, Cancer registry, Radiofrequency radiation

## Abstract

**Purpose:**

The association between the use of mobile phones use and the risk of brain cancer remains controversial. The aim of this study is to describe trends in the incidence of central nervous system (CNS) cancers in Spain and its possible relationship with mobile phone use.

**Methods:**

Trends and trend changes from 1985 to 2015 in adjusted incidence rates of CNS cancers by sex, age (adults and children), site, and histological type were assessed using data from 14 general and paediatric cancer registries.

**Results:**

The study included 20,325 CNS malignancies in adults and 2,372 in children. For adults, the overall rate of malignant brain tumours showed a slight increase of 0.2% (95% CI: 0.1 - 0.4) per year. This increase was concentrated in the early years up to 1996 (1.7% per year, 95% CI: 0.9 - 2.6) followed by 20 years of a non-significant slight decline of -0.1% (95% CI: -0.4 - 0.1) per year until 2015. In children, an increase of 7.6% (95% CI: 2.4 – 13.1) per year until 1991 followed by a decrease of -1.0% (95%CI: -1.7 - -0.3) per year until 2015 was observed. This increase in the incidence in 1980s and early 1990s could be explained by diagnostic improvements especially in imaging techniques implemented during these years.

**Conclusion:**

The present findings do not support a possible relationship between the use of mobile phones and the incidence of malignant brain tumours. However, the possibility of the presence of a weak correlation or that a longer latency period would be needed to observe a possible ecological correlation cannot be discarded.

**Supplementary Information:**

The online version contains supplementary material available at 10.1007/s12094-025-03932-y.

## Introduction

The aetiology of brain cancers is poorly understood [1, 2] and the only well-established risk factors, — ionizing radiation [2] and rare hereditary syndromes [3]—, account for a small proportion of brain tumours. Radiofrequency electromagnetic fields (RF-EMF) emitted from mobile phones (MPhs) were proposed as a risk factor for brain tumours [4]. When a MPh is attached to the head, the brain is exposed to high levels of radiofrequency (RF) radiation [5]. Thus, there is concern about a possible association with brain cancer [6].

In 2011 the International Agency for Research on Cancer (IARC) classified RF-EMF as “possibly carcinogenic to humans” based above all on one cohort study and five case–control studies [7]. A recent systematic review and meta-analysis found that for near field RF-EMF exposure to the head from mobile phone use, there was moderate certainty evidence that it likely does not increase the risk of glioma and meningioma in adults, or of paediatric brain tumours [8].

Given that the vast majority of the population regularly uses MPhs, even a relatively small excess risk would result in a significant number of additional cases of brain tumours, and such an increase would be observable in cancer surveillance systems [9]. The World Health Organisation (WHO) identified as a high research priority the monitoring of brain tumour incidence trends through population-based cancer registries combined with population exposure data [10]. Since then, ecological studies have shown that, although the prevalence of MPh use has increased massively, no indication of any increase related to the use of mobile phones has been observed [11–15]. However, it has been suggested that the introduction of better diagnostic imaging methods such as computed tomography (CT) and magnetic resonance imaging (MRI) improved the detection of brain cancers, leading to a higher incidence, especially all in old ages [16].

The use of MPhs has grown dramatically. In Spain, for example, only 2% of the population had a MPh in 1995, whereas there were approximately 33.5 million MPhs in 2002, about 42.7 million in 2005 and more than 51 million in 2009. Between 2010 and 2015, the figure fluctuated between 51 and 52 million [17].

The “Strategic Plan for Health and Environment” of the Spanish Ministry of Health [18] proposed different lines of research, with monitoring of brain tumour rates and trends in the Spanish population as one of the actions. In accordance with the objective of the Spanish Ministry of Health, the Spanish Network of Cancer Registries (REDECAN) has analysed trends in the incidence rates of central nervous system (CNS) tumours, by sex, age (adults and children), site of the primary tumour and histological type during the period 1985–2015.

## Methods

Two separate analyses for the period 1985 and 2015 have been carried out, one in adults (aged 15 years and older) and the other in children (0 to 14 years). Incidence data for primary malignant CNS tumours diagnosed in adults were obtained from 12 Spanish population cancer registries covering 12 provinces and 3 islands. Incidence data for childhood were obtained from 12 general population cancer registries, from a childhood population tumour registry and from the Spanish Registry of Childhood Tumours. These databases cover a total of 20 provinces and 3 islands. Table 1 shows the participating registries, as well as the territory (province/s or island/s) covered by each of them, and the period of data included in the analysis of each of them for each age group (adults and children). The proportion of population covered with respect to the total population of Spain varies depending on the year. In the years with more extensive coverage, this proportion was 24% for adults and 62.5% for children.

**Table 1:** Spanish cancer registries contributing data to the study of trends in adult and in children brain cancers

Registry	Province/island	Period covered	
		Adults	Children (0–14)
Asturias	Asturias	1991–2013	1991–2013
Canarias	Gran Canaria, Tenerife (islands)	1993–2015	1993–2015
Castellón	Castellón	2004–2015	
Castilla y León	Salamanca	2011–2015	2011–2015
Euskadi	Álava, Guipúzcoa, Vizcaya	1986–2015	1986–2015
Girona	Girona	1994–2015	1994–2015
Granada	Granada	1985–2015	1985–2015
La Rioja	La Rioja	1993–2014	1993–2014
Mallorca	Mallorca (island)	1988–2013	1988–2013
Murcia	Murcia	1985–2015	1985–2015
Navarra	Navarra	1985–2015	1985–2015
Tarragona	Tarragona	1985–2015	1985–2015
Comunitat Valenciana	Alicante, Castellón, Valencia		1985–2015
Spanish Registry of Childhood Tumours (RETI)	Barcelona, Huesca, Lleida, Madrid, Teruel, Zaragoza		2000–2015

These registries provided data of the subsite (codes C70.0–C72.9 of the International Classification of Diseases for Oncology, 3rd edition, first revision -ICD-O-3.1-) [19], the histological type (morphological codes of the ICD-O-3.1) [19], the sex, the year of diagnosis and the age at diagnosis.

The study included a general analysis for all CNS malignant tumours and specific analyses by subsite and histological type. Table 2 presents the topographies and morphologies and the groupings used in the analyses. Due to the variability amongst registries in the registration of CNS tumours regarding the inclusion of benign tumours and tumours of uncertain behaviour, tumours of malignant behaviour (code 3 of the ICD-O-3.1) were only included in the present study [19]. Granular cell tumours, alveolar soft tissue sarcomas and haematological tumours were not included in any category.

**Table 2:** Topographies and histological types included in the analyses

Description	Codes
CNS^a^	C70–C72
Topographies	
Meninges	C700–C701, C709
Brain	C710–C719
Frontal	C711
Temporal	C712
Parietal	C713
Occipital	C714
Others	C710, C715–C717
Overlapping	C718
Unspecified	C719
Histologies	
Global specified ^b^	8010–9561
Gliomas and embryonal tumours^c^	9380–9480
Glioblastoma	9440–9442
Meningioma	9530–9539
Other^d^	8010–9371, 9490–9508, 9540–9561
Unspecified^e^	8000–8004

^a^*CNS* Includes all tumours of the central nervous system (C70 meninges, C71 brain, C72 spinal cord, cranial nerves, and other parts of the central nervous system

^b^Global specified: Includes all tumours, except tumours with non-specified histology

^c^Gliomas and embryonal tumours: Includes astrocytomas, ependymomas, other gliomas, choroid plexus tumours, and embryonal tumours except those included in “Other” (medulloepithelioma, neuroepithelioma, spongioneuroblastoma, and atypical teratoid/rhabdoid tumour)

^d^Other: Includes: All tumours, except those included in gliomas and embryonal tumours, neuroepitheliomatous neoplasms, nerve sheath tumours and tumours with non-specific histology. Also includes neuroepitheliomatous neoplasms and nerve sheath tumours

^e^Unspecified: Includes Neoplasms without specific histological specification

Population estimates as of July 1 of each year during the study period for each province or island with cases included in the study were obtained from the National Institute of Statistics (INE).

Incidence in adults and children have been analysed separately. Regarding sex, the two sexes have been analysed together and separately. First, the study has analysed all CNS malignant tumours as a whole (C70, C71 and C72). By topography, the study has analysed malignant tumours of the meninges as a whole (C700 to C709) and non-meningeal malignant tumours of the brain as a whole (C710–C719) and by subsite (frontal lobe, temporal lobe, parietal lobe, occipital lobe, other subsites, contiguous overlapping lobe, and non-specific site). Neoplasms of the spinal cord, cranial nerves and other parts of the CNS (ICD-10 code C72) have not been specifically analysed.

Based on the histology of tumours, those with specified histology (Global specified) and those of unknown histological type (Unspecified) were analysed separately. Within the tumours with specified histology, Gliomas and embryonal tumours, Meningiomas (only malignant) and Other histological types as a whole have been analysed separately according to the WHO Classification [1]. Furthermore, a specific analysis of glioblastoma was performed.

Age-standardized annual incidence rates (ASIR) per 100,000 person-years for adulthood and per 1,000,000 children-years for childhood were calculated using the 2013 standard European population.

### Statistical analysis

Trend changes in age- and registry-adjusted incidence rates during the study period were assessed for both sexes as a whole and separately for each sex using point-change Poisson models [20]. This analysis provides an asymptotic test for the existence of a change point, the estimate and the 95% confidence interval (CI) for the timing of the change point, and the estimates and 95% CIs for annual percentage changes (APC) in incidence rates for the entire period 1985–2015 and in the incidence rates before and after the point of change. This method was chosen rather than a trend analysis of pre-determined periods because it allowed a better observation of the reality of the evolution of incidence over the 31 years covered by the study. On the other hand, if there was really a causal association between MPhs use and the development of malignant brain tumours, since the latency period(s) of each type of tumour was unknown, it was impossible to assign the time that would define with certainty the onset of the effects of this association.

The graphical representations of the time trends showed the year-to-year evolution of the 2013 European standard population-adjusted incidence rates, smoothed by calculating the five-year moving average, i.e. including 5 years, 2 before and 2 after the year of interest.

The fits of the Poisson change-point models were performed through the R software using functions for fitting change-point models that are available in the supplementary material of the article by Pollán et al. [21].

## Results

### Incidence trends in adults

The study population of adults included 20,325 malignant CNS tumours (11,217 in men and 9,108 in women) diagnosed in different territories and periods between 1985 and 2015 (Tables 3, S1 and S2). Of these, 72.3% were gliomas and only 1.4% were malignant meningiomas. A total of 25.3% of cases were tumours of unspecified histology. The most common lobar specific topographies of non-meningeal tumours were the frontal lobe (17.6%), and the temporal lobe (16.6%). There were 20.4% tumours in other sites, 22.9% in overlapping sites and 9.6% with an unspecified site (Table 3).

**Table 3:** Temporal trends in the incidence of central nervous system cancers in adults, both sexes. Spain, 1985–2015

Tumoral type	*N*	1985–2015		Join point	Period 1		Period 2		*p* value
		APC	(95%CI)		APC	(95%CI)	APC	(95%CI)	
CNS	20,325	**0.3**	**(0.1–0.5)**	**1995.7**	2.0	(1.2**–**2.9)	– 0.1	(– 0.4 to 0.1)	**0.000**
Topography									
Meninges	392	0.3	(– 0.7 to 1.2)	2007.5	1.6	(0.2**–**3.0)	– 4.1	(– 7.4 to – 0.6)	0.113
Brain	19,436	**0.2**	**(0.1–0.4)**	**1995.8**	1.7	(0.9**–**2.6)	– 0.1	(– 0.4 ro 0.1)	**0.003**
Frontal lobe	3,427	**2.6**	**(2.2–3.1)**	1995.1	4.1	(1.8**–**6.4)	2.4	(1.8**–**3.0)	1.000
Temporal lobe	3,217	**1.9**	**(1.5–2.3)**	2000.3	2.6	(1.4**–**3.7)	1.5	(0.7**–**2.3)	1.000
Parietal lobe	1,958	** – 0.9**	**(– 1.3 to – 0.4)**	**1994.2**	7.0	(4.2**–**10.0)	– 2.1	(– 2.8 to – 1.5)	**0.000**
Occipital lobe	549	– 0.4	(– 1.2 to 0.3)	2005.5	0.8	(– 0.5 to 2.1)	– 3.0	(– 5.3 to – 0.7)	0.195
Others brain	3,974	** – 2.0**	**(– 2.4 to – 1.5)**	2005.7	– 1.4	(– 2.1 to – 0.8)	– 3.2	(– 4.5 to – 1.9)	0.418
Contiguous sites	4,442	**0.8**	**(0.4–1.2)**	2010.5	1.1	(0.6**–**1.6)	– 1.7	(– 4.2 to 0.9)	0.449
Brain unspecified	1,869	** – 2.9**	**(– 3.4 to – 2.4)**	**2003.9**	1.0	(– 0.1 to 2.0)	– 9.5	(– 11.0 to – 7.9)	**0.000**
Histology									
Global specified	15,187	**2.0**	**(1.7–2.2)**	**1998.6**	4.5	(3.7**–**5.4)	0.8	(0.4**–**1.2)	**0.000**
Gliomas and embryonal t	14,693	**2.0**	**(1.7–2.2)**	**1998.8**	4.3	(3.5**–**5.1)	0.9	(0.5**–**1.3)	**0.000**
Glioblastoma	6,723	**4.1**	**(3.8–4.5)**	**2003.5**	5.3	(4.5**–**6.0)	3.0	(2.3**–**3.7)	**0.007**
Meningiomas	283	0.5	(– 0.5 to 1.6)	2007.3	1.6	(0.0**–**3.2)	– 2.9	(– 6.6 to 0.9)	0.488
Others	211	– 0.6	(– 1.7 to 0.6)	1994.4	9.5	(1.4**–**18.2)	– 1.8	(– 3.2 to – 0.3)	0.082
Unspecified	5,138	** – 4.2**	**(– 4.5 to – 3.9)**	**1993.6**	– 1.9	(– 3.5 to – 0.2)	– 4.7	(– 5.1 to – 4.2)	**0.036**

*APC* Annual Percentage Change of the Adjusted Rate. *Join point* Change point (JoinPoint) that determines Periods 1 and 2

The p value indicates the level of statistical significance of the change in trend between the 2 periods. Bold indicates statistical significance (*p* < 0.05)

For descriptive purposes, Table 4 presents the ASIR for the biennia 1985–1986, 2000–2001 and 2014–2015 for adults and for children separately. In men, the ASIR of all CNS tumours in 1985–1986 was 9.7 and 9.8 in 2014–2015. In women, these rates were 7.0 and 7.8, respectively. The incidence rates of gliomas increased in both men (4.7–8.4) and women (3.8 and 6.5) whilst those of non-specific histological types decreased by similar magnitudes (Table 4).

**Table 4:** Age-adjusted incidence rates of central nervous system cancers by sex in adults and children. Spain, biennia 1985–1986, 2000–2001 and 2014–2015

Tumoral type	Men			Women			Boys			Girls		
	1985	2000	2014	1985	2000	2014	1985	2000	2014	1985	2000	2014
	1986	2001	2015	1986	2001	2015	1986	2001	2015	1986	2001	2015
CNS	9.7	10.2	9.8	7.0	7.7	7.8	24.1	25.3	24.0	20.1	20.1	27.1
Meninges	0.1	0.2	0.1	0.1	0.2	0.1						
Brain	9.5	9.8	9.4	6.8	7.3	7.5	22.3	23.0	19.8	18.3	17.0	22.9
Glioma	4.7	7.4	8.4	3.8	5.3	6.5	21.7	23.8	21.7	17.4	18.1	24.5
Meningioma	0.1	0.1	0.1	0.1	0.1	0.0						
Others	0.0	0.2	0.2	0.0	0.1	0.1	0.8	0.9	1.4	0.8	0.4	2.0
Unspecified	5.0	2.5	1.1	3.1	2.2	1.2	1.6	0.7	0.7	2.0	1.6	0.6

Adults: Age-adjusted incidence rates for 100,000 person/years using the 2013 European standard population

Children: Age-adjusted incidence rates for 1,000,000 person/years using the 2013 European standard population

For the overall population (men and women), the rate of malignant brain tumours in the whole period between 1985 and 2015 showed a slight increase of 0.2% per year (95% CI 0.1–0.4). However, change point analysis showed that this increase was concentrated in the early years (1.7% per year, 95% CI 0.9–2.6) up to 1996 followed by a slight decrease of – 0.1% (95% CI – 0.4 to – 0.1) per year up to 2015 (Table 3). By sex, there was stability in men (Table S1). In women, an increase of 0.6% (95% CI 0.3–0.8) per year was observed throughout the entire period, although this increase was concentrated in the early years up to 2000 (1.9% per year, 95% CI 1.1–2.7) followed a period of stability (– 0.2% per year, 95% CI – 0.7 to 0.3) up to 2015 (Table S2).

By subsites, there was a decrease, overall and by sex, in the rates of Other brain, Non-specific brain and Parietal Lobe. Occipital lobe incidence rates showed a statistically significant decrease in males and stability in females. On the contrary, the incidence rates in the frontal and temporal lobes showed increases throughout the overall period but more marked during the first years (Table 3, S1 and S2).

Malignant tumours of the meninges were very rare. In both sexes, there was stability (annual increase of 0.3% not statistically significant, 95% CI – 0.7 to 1.2) throughout the study period (Table 3). In men, a decrease of – 1.9% (95% CI – 3.1 to – 0.6) per year was observed, whereas there was a statistically non-significant decrease of – 0.3% per year (95% CI – 1.5 to 1.0) in women (Tables S1 and S2).

By histological type, the rates of gliomas showed significant increases in both sexes, more notably in the early period up to the late 1990 s. The same findings were observed for glioblastoma, the most frequent type of glioma. In contrast, the rates of cancers with Unspecified histology showed a statistically significant sharp decrease in both sexes (– 4.2%, 95% CI – 4.5 to – 3.9), as well as in men and women separately with an APC of – 4.7% (95% CI – 5.1 to – 4.3) in men and – 3.2% (95% CI – 3.6 to – 2.8) in women. This decrease was more marked during the second period, from 1990 onwards in men and from 1999 onwards in women (Table 3, S1 and S2).

**Fig. 1:**
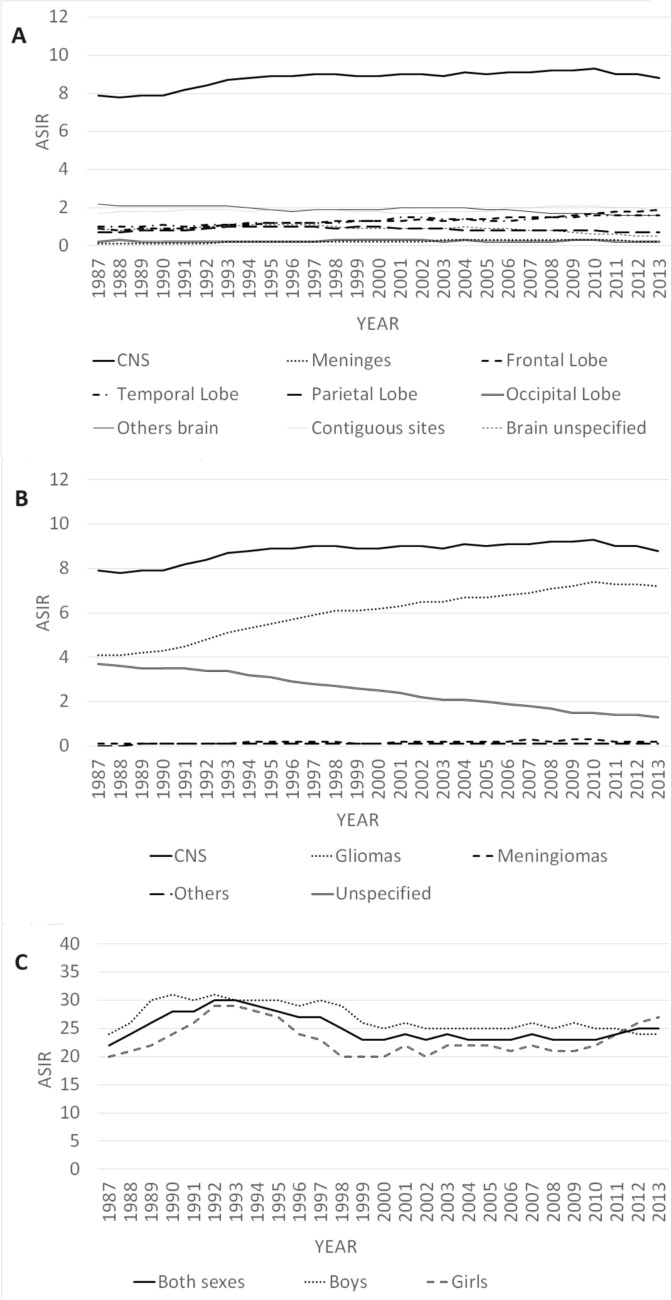
**A** Evolution of smoothed incidence rates adjusted to the standard European population of malignant tumours of the central nervous system according to tumour subsite in adults. Spain. 1987–2013. **B** Evolution of smoothed incidence rates adjusted to the standard European population of malignant tumours of the central nervous system according to major histological groups in adults. Spain, 1987–2013. **C** Evolution of smoothed age-adjusted incidence rates of malignant tumours of the central nervous system in children. Spain, 1985–2015. Age-standardized incidence rates per 1,000,000 children/years using the standard European population of 2013

Figure 1A shows the slight increase in global rates of CNS malignancies, concentrated in the first half of the 1990 s, and Fig. 1B the increase in gliomas and the decrease of a similar magnitude in tumours of unspecified histology. Figures S1 and S2 show the same data by sex.

### Incidence trends in childhood

The study of the paediatric population included a total of 2372 CNS malignancies (1275 in boys and 1097 in girls). Of these, 89.3% were gliomas and embryonal tumours, 0.4% malignant meningiomas, 5.8% other histological types and 4.5% tumours of unspecified histology. The most frequent lobar-specific anatomical subsite of non-meningeal tumours was the temporal lobe (5.2%), followed by the frontal lobe (4.2%), parietal lobe (2.8%) and occipital lobe (1.3%). There were 75.4% tumours in other locations, 3.0% in overlapping sites and 8.1% with an unspecified site (Table 5).

**Table 5:** Temporal trends in the incidence of malignant tumours of the central nervous system in children. Spain, 1985–2015

Tumoral type	*N*	1985–2015		Join	Period 1		Period 2		*p* value
		APC	(95%CI)	point	APC	(95%CI)	APC	(95%CI)	
CNS	2372	0.0	(– 0.6 to 0.6)	**1991.2**	6.8	(2.0–11.9)	– 0.6	(– 1.3 to 0.1)	**0.040**
Topography									
Meninges	11				Not evaluable				
Brain	2,063	−0.3	(– 0.9 to 0.3)	**1991.1**	7.6	(2.4–13.1)	– 1.0	(– 1.7 to − 0.3)	**0.019**
Frontal lobe	86	0.3	(– 2.3 to 3.0)	2011.4	− 0.5	(– 3.7 to 2.7)	9.6	(– 9.7 to 33.0)	1.000
Temporal lobe	107	0.3	(– 1.9 to 2.6)	1995.7	3.9	(−5.4 to 14.0)	– 0.7	(−4.1 to 2.8)	1.000
Parietal lobe	57	0.2	(– 3.5 to 4.1)	1994.4	9.3	(– 4.3 to 24.9)	– 2.5	(−7.6 to 2.9)	0.909
Occipital lobe	26				Not evaluable				
Others brain	1556	– 0.3	(– 0.9 to 0.4)	1991.4	6.4	(0.7–12.3)	– 0.8	(– 1.7 to 0.0)	0.157
Contiguous sites	63	0.4	(– 2.6 to 3.5)	1996.0	4.9	(– 6.4 to 17.5)	– 1.0	(– 5.5 to 3.7)	1.000
Brain unspecified	168	– 0.3	(– 2.0 to 1.5)	1997.3	3.6	(– 2.2 to 9.7)	– 2.1	(– 5.1 to 1.0)	1.000
Histology									
Global specified	2264	0.2	(– 0.4 to 0.8)	1991.1	6.8	(1.7—12.2)	– 0.3	(– 1.0 to 0.4)	0.082
Gliomas and embryonal t	2118	– 0.1	(– 0.6 to 0.5)	1991.4	6.0	(1.2—11.0)	– 0.6	(– 1.3 to 0.1)	0.101
Glioblastoma	62	1.3	(– 2.4 to 5.1)	2013.7	– 0.3	(– 4.3 to 3.9)	47.7	(– 4.4 to 128.1)	0.639
Meningiomas	9				Not evaluable				
Others	137	1.2	(– 1.2 to 3.7)	2010.6	2.6	(– 0.6 to 5.9)	– 7.4	(– 18.9 to 5.7)	1.000
Unspecified	108	– 0.4	(– 2.8 to 2.1)	2008.5	0.0	(– 3.2 to 3.4)	– 2.6	(– 14.6 to 11.0)	1.000

*APC* Annual Percentage Change of the Adjusted Rate. *Join point* Change point (JoinPoint) that determines Periods 1 and 2

The *p* value indicates the level of statistical significance of the change in trend between the 2 periods. Bold indicates statistical significance (*p* < 0.05)

In boys, the ASIR for all CNS tumours in the 5-year period between 1985 and 1989 was 24.1 and in the 5-year period between 2011 and 2015 was 24.0. In girls, these rates were 20.1 and 27.1, respectively. The incidence rates of gliomas in boys were 21.7 in both 5-year periods and in girls, they were 17.4 and 24.5, respectively. The incidence rates for cancers of non-specific histology decreased from 1.6 to 0.7 in boys and from 2.0 to 0.6 in girls (Table 4).

In the overall study period for boys and girls combined (Table 5), no significant trend was observed for either the CNS tumour group or any of the groups studied. However, using the change point model, an increase in brain tumours of 7.6% (95% CI 2.4–13.1) per year until 1991 was observed followed by decrease of – 1.0% (95% CI – 1.7 to – 0.3) per year until 2015.

In boys, the overall rate of malignant brain tumours for the whole study period remained stable, although it showed an increase of 39.5% (95% CI 8.7–79.0) per year until 1987, followed by a significant decrease of – 1.3% (95% CI – 2.3 to – 0.3) per year until 2015. In girls there was also a stability in the rates of malignant brain tumours throughout the overall study period, with an almost significant increase of 7.7% (95% CI – 0.2 to 16.2) per year until 1991 and stability until 2015 (Tables S3 and S4).

Figure 1C shows an increase in children incidence rates between 1987 and 1991–1992, followed by a decrease until the late 1990 s and stability until the end of the study period.

## Discussion

In Spain, the use of MPhs grew explosively in the decade around 2000 and under the assumption that this could be a cause of brain cancer, the effects of such dramatic exposure could be seen in incidence rates in the general population, unless the induction period was very long or limited to very long-term users. In Spain, the massive deployment of mobile telephony took place in the second half of the 1990 s and the first half of the 2000 s. In 2005, there were already almost 43-million MPh lines. Therefore, the period 1985–1995 could be considered as the period prior to the massive use of mobile telephony, the period 1995–2005 as the period of mass deployment of mobile telephony and the period 2005–2015 as the first full decade of mass use of mobile telephony. This study analysed, for the first time, the trends in the incidence of brain malignant tumours in our country, globally and by topographical and morphological subtype for the period 1985–2015, to determine whether it was possible to hypothesise a relationship between the increased use of MPhs and the evolution of these trends.

In adults, there was an increase in the incidence of brain tumours in the first 10 years of the study period followed by a slight non-significant decrease during the following 20 years that can be interpreted as a period of incidence stability. The increasing incidence of brain cancer observed in adults in the early years of the study period is consistent with the period of improvement in diagnostic techniques in many countries in the 1980 s and 1990 s, largely explained by advances and widespread use of CT and MRI in routine clinical practise [22–24]. Regarding childhood tumours and for the whole study period, no statistically significant trends in any of the tumour types were observed, although there was an increase in the rates only up to 1991–1992, also probably related to diagnostic improvements occurring during these years.

Of note is the large number of tumours with unspecified categories, both in terms of subsite and, above all, histological type. The incidence rates of gliomas rose sharply until 1999 and continued to increase but less markedly thereafter. Glioblastoma also showed a greater increase in the early years. In parallel, there was a strong downward trend in the incidence of brain malignancies of unspecified histology throughout the overall study period, particularly from 1990 onwards in men and from 2000 onwards in women. In the United States, Jukich et al. [25] observed increases in incidence for glioblastoma, oligodendrogliomas and astrocytomas, and decreases for NOS, astrocitoma NOS and glioma NOS and concluded that improvements in diagnostic technology, in addition to changes in international classification and coding systems, were likely responsible for the observed decreases in the incidence of NOS subgroups and corresponding increases in glioma subgroups [26].

Regarding the subsite, rates increased in the frontal lobe, in the temporal lobe (which receives the highest radiofrequency dose from MPh use) and in contiguous sites. In contrast, cancers of the parietal lobe decreased from 1994 onwards (since 2001 in men and 1994 in women), and more markedly, those of the “Other brain” site and especially those of the “Brain unspecified” globally and in both sexes separately.

Many studies on brain tumour trends also reported a high proportion of cases with unspecified subsite and histology, making it difficult to analyse trends by topographical and morphological subgroups. In England, de Vocht et al. [27]. did not find significant trends in the overall incidence of brain tumours for any specific age group between 1998 and 2007 but increases in the rates for cancers of the temporal lobe in men and women were observed, as well as decreases in the rates of tumours of the parietal lobe, cerebrum and cerebellum in men. According to this study, the increased use of MPhs between 1985 and 2003 has not led to a noticeable change in the incidence of brain cancer in England between 1998 and 2007. Un update of this study covering the period 1996 to 2017 continues to provide little evidence of an association with MPh use [14]. In New Zealand, no consistent increases in the rates of all primary brain cancers were found between 1995 and 2010, but there was a significant decrease in the incidence of brain cancers in subjects younger 70 years associated with an increase in gliomas in over 70 years of age. It was concluded that the increase in gliomas in the population over 70 years of age was probably due to improvements in diagnosis [16]. Extension of this study to 2020 shows a small decrease in the incidence of gliomas in ages 10 to 69 years and in brain subsites receiving more RF energy, but an increase in ages over 80 consistent with improved diagnostic methods [13]. In Australia, there was an increase in the incidence of temporal lobe gliomas during the period of increased use of CT and MRI diagnostic studies (1982–1992), which was also observed, to a lesser extent, in the period with advances in MRI use (1993–2002). In the period with substantial MPh use (2003–2013), no increase in temporal lobe gliomas was observed, although there was a sharp decline in gliomas of unspecified site [27]. Also in England, glioblastoma and meningioma rates increased in men and women between 1995 and 2013. The incidence of unclassified brain tumours declined between 1995 and 2007 and remained stable thereafter. The authors concluded that part of the increase in the incidence of major subtypes could be explained by advances in clinical practise and improved cancer registration practises [28]. In the Nordic countries, as in Spain, the use of MPhs increased sharply in the mid-1990 s. From 1974 to 2003, the incidence rate of glioma increased, but no changes in incidence were observed between 1998 and 2003, the time at which possible associations between MPh use and cancer risk would be informative over an induction period of 5–10 years [29]. In Finland, no increase in the incidence of malignant gliomas was observed between 1990 and 2006, but in the older age group, an increase in the incidence of malignant gliomas and a decrease in unspecified brain tumours was found [30]. In the United States, between 1977 and 2006, using data from the Surveillance, Epidemiology and End Results (SEER) programme no increases were observed in cancers of the temporal or parietal lobe, nor in cancers of the cerebellum, the parts of the brain that would be most exposed to RF radiation from MPhs. Based on these data, the hypothesis that the use of MPhs causes brain cancer was not supported [11]. In summary, most studies that have analysed trends in brain tumour incidence did not find an increase in brain tumours following the massive introduction of MPh use but find an increase in specified brain tumours, such as gliomas, along with a decrease in unspecified tumours.

For a relationship between the use of MPhs and brain cancer to be causal, one would expect an increasing trend in the period after the mass deployment of MPhs and no trend in the earlier periods. However, in our study the opposite is observed. The incidence trend of gliomas rose with some intensity until 1999 and, from this moment, it increased very slightly until the end of the study period. As in the aforementioned studies, this coincides with a decrease in the incidence rates of tumours with non-specific histology whose intensity of decrease, in our study, was maximum from 1991 in men and from 2000 in women.

During the 1990 s and early 2000 s, technological improvements in imaging methods (CT and especially MRI) led to significant improvements in the discrimination of brain tumour types and subtypes. As consequence of these improvements, increases in certain subtypes were accompanied by decreases in unspecified subtypes, whilst the overall incidence of brain tumours remained stable. Several studies suggested that improvements in imaging methods were responsible for the upward trends in the diagnosis of certain brain subtypes. Other factors, such as improved access to health care and an increase in the number of specialists, may also have played a role in the rising incidence.

Although when using MPhs, the temporal and parietal lobes are more exposed to RF-EMF than other sites such as the frontal lobe, overall, the increase in frontal lobe gliomas was higher than that of temporal lobe gliomas. As mentioned above, there was a large number of gliomas from unspecified and overlapping locations in our data and, in turn, a significant decrease in these over the period. This led to an increase in the incidence rates of the specified sites. A similar phenomenon occurred with the rates by histological type, which would explain the increases in gliomas, particularly glioblastomas. This fact, which is at the root of the difficulties in interpreting past incidence trends, also has a positive side; monitoring trends in these tumours may become increasingly accurate in the future.

The present study has some limitations. First, the incomplete level of national coverage of cancer registries, especially in adults, as the coverage of cancer registries in Spain is partial. However, information was available for the entire population covered by cancer registries during the period under study. Second, the study follow-up extends only until 2015 to ensure data availability in most cancer registries. Third, the variable representativeness over time, with fewer registries in the early years (1980 s and 1990 s), so that data are not fully comparable over the study period although registry-adjusted incidence rates have been used. Moreover, the quality of cancer registration methods in the early years might be different and tentatively lower than in recent periods. However, all population-based cancer registries that have participated in the study follow the international criteria defined by the IARC for cancer registries and, consequently, the completeness of cases in their geographical area is very high. In addition, the completeness of the casuistry of childhood cases in the six provinces provided by the Spanish Registry of Childhood Tumours is also very high. Another limitation is not being able to include benign tumours and tumours with uncertain behaviour in the study due to the heterogeneity in the registration practices of these tumours.

Likewise, information on the use of MPhs is limited and information on the number of MPh lines over time may not be an indicator of actual use. Furthermore, the quantification of exposure remains unclear, and it is likely that individual patterns of MPh use have changed over time and that changes in technology have occurred. Furthermore, both the biology of brain cancers and their aetiology, and consequently latency periods, are poorly understood. On the other hand, whilst ionising radiation has been shown to induce brain cancer by causing DNA damage with a latency period of approximately 5 years or more [31], RF-EMF exposure occurs via non-ionising radiation that does not cause direct DNA damage. A possible increased risk of brain cancer could have both a shorter latency of less than 5 years or a longer latency, indicating both its effect on tumour initiation and promotion, respectively. In this respect, to determine from which point onwards a period of time should be considered as the period after MPh use, remains difficult to ascertain.

Finally, although this is an appropriate observational study to determine trends, it is possible that a stable trend in brain tumour incidence would have masked a true increase in risk related to the use of MPhs if some other risk factor had caused a decrease in the risk.

## Conclusions

The results of the present study show an upward trend in the incidence of malignant brain tumours in the 1980 s and early 1990 s, and a subsequent stabilisation in both adults and children. This increase could be due to the diagnostic improvements based mainly on the progress of imaging techniques implemented in clinical practise during 1980 s and 1990 s. These results do not support the hypothesis of a possible correlation between the use of MPhs and the incidence of malignant brain tumours. However, the possibility that a weak correlation exists or that a longer latency period is needed to hypothesise a possible ecological correlation cannot be excluded. Nevertheless, it is important to continue analysing trends in the incidence of brain tumours globally and according to histological and anatomical categories. Improving the quality of diagnoses could facilitate the detection of a possible increase in incidence in the future, which would suggest a relationship with possible environmental factors, including the increase in the use of MPhs in recent decades.

## Supplementary Information

Below is the link to the electronic supplementary material.

Supplementary file1 (DOCX 161 KB)

## Data Availability

The data from this research is not shared.
