# Supplementary material for: Trends in the incidence of brain cancer and the use of mobile phones: analysis of the Spanish Network of Cancer Registries (REDECAN)
Source: Clin Transl Oncol. 2025 May 14;27(11):4221–31. doi: 10.1007/s12094-025-03932-y (PMC12559055; doi:10.1007/s12094-025-03932-y)
Supplement: Supplementary file 1 — Supplementary file1 (DOCX 161 KB) [file 12094_2025_3932_MOESM1_ESM.docx]

**Trends in the incidence of brain cancer and the use of mobile phones: Analysis of the Spanish Network of Cancer Registries (REDECAN)**

**Authors:**  Jaume Galceran^1,2^, Alberto Ameijide^1,2^, Adela Cañete^3,4,5^, Rafael Peris-Bonet^3^, Arantza López de Munain^6^, Amaia Aizpurúa^6^, Marta de la Cruz^6^, Arantza Sanvisens^7,8^, María José Sánchez^9,10,11,12^, Isabel Palacios^13^, Paula Franch^14,15^, Antonia Sánchez^16^, Marcela Guevara^17,18^, Marià Carulla^1,2^, Pilar Gutiérrez^19^, Isabel Sáez^20^, Marta Rodríguez^21^, Araceli Alemán^22^, Consol Sabater^23^ on behalf of Spanish Network of Cancer Registries (REDECAN).

1. Tarragona Cancer Registry, Cancer Epidemiology and Prevention Service, Sant Joan de Reus University Hospital, Tarragona, Spain.
2. Pere Virgili Health Research Institute (IISPV), Reus, Tarragona, Spain.
3. Paediatric Oncohematology Unit, Hospital La Fe, Valencia, Spain.
4. Spanish Registry of Childhood Tumours (RETI-SEHOP), Universitat de Valencia, Valencia, Spain.
5. Department of Paediatrics, University of Valencia, Valencia, Spain.
6. Basque Country Cancer Registry, Department of Health, Basque Government, Vitoria-Gasteiz, Spain.
7. Epidemiology Unit and Girona Cancer Registry, Oncology Coordination Plan, Catalan Institute of Oncology, Girona, Spain.
8. Girona Biomedical Research Institute Dr. Josep Trueta (IDIBGI), Girona, Spain.
9. Granada Cancer Registry, Andalusian School of Public Health (EASP), Granada, Spain.
10. Instituto de Investigación Biosanitaria Ibs.GRANADA, Granada, Spain.
11. Department of Preventive Medicine and Public Health, University of Granada, Granada, Spain.
12. Consortium for Biomedical Research in Epidemiology and Public Health (CIBERESP).
13. La Rioja Cancer Registry, Epidemiology and Health Prevention Service, Logroño, Spain.
14. Mallorca Cancer Registry, Public Health and Participation Department, Palma de Mallorca, Spain.
15. Health Research Institute of the Balearic Islands (IdISBa), Palma de Mallorca, Spain.
16. Murcia Cancer Registry, Department of Epidemiology, Regional Health Authority, Instituto Murciano de Investigación Biosanitaria (IMIB)-Arrixaca, Murcia University, Murcia, Spain.
17. Navarra Cancer Registry, Navarra Public Health Institute, Pamplona, Spain.
18. Epidemiology and Public Health Area, Navarra Institute for Health Research (IdiSNA), Pamplona, Spain.
19. Castilla y León Cancer Registry, Public Health Directorate, Castilla y León Government, Valladolid, Spain.
20. Registry of Childhood and Adolescent Tumours of the Valencian Community, Generalitat Valenciana, Valencia, Spain.
21. Asturias Cancer Registry, Public Health Directorate, Asturias, Spain.
22. Canary Islands Cancer Registry, Public Health Directorate, Canary Islands Government, Tenerife, Spain.
23. Castellón Cancer Registry, Directorate General of Public Health and Addictions, Valencian Government, Castellón, Spain.

**Corresponding author:** Jaume Galceran ([jaume.galceran@salutsantjoan.cat](mailto:jaume.galceran@salutsantjoan.cat))

**Supplementary material**

**Table S1** Time trends in the incidence of central nervous system cancers in adult men. Spain, 1985-2015

|  |  | **1985-2015** | | **Join** | **Period 1** | | **Period 2** | | **P value** |
| --- | --- | --- | --- | --- | --- | --- | --- | --- | --- |
| **Tumoral type** | **N** | **APC** | **(95%CI)** | **point** | **APC** | **(95%CI)** | **APC** | **(95%CI)** |  |
| CNS | 11,217 | 0.0 | (-0.3 - 0.2) | 2011.4 | 0.2 | (- 0.1 - 0.6) | -3.0 | (-5.2 - -0.8) | 0.070 |
| **Topography** | | | | | | | | | |
| Meninges | 188 | **-1.9** | **(-3.1 - -0.6)** | 2012.9 | -2.1 | (-3.6 - -0.7) | 5.4 | (-13.0 - 27.7) | 1.000 |
| Brain | 10,763 | -0.1 | (-0.3 - 0.2) | 2011.8 | 0.2 | (-0.2 - 0.5) | -3.3 | (-5.8 - -0.7) | 0.122 |
| Frontal lobe | 1,830 | **2.3** | **(1.7 - 2.8)** | 2014.0 | 2.5 | (1.9 - 3.2) | -9.1 | (-20.2 - 3.4) | 0.530 |
| Temporal lobe | 1,922 | **1.4** | **(0.9 - 1.9)** | 2007.4 | 0.9 | (0.1 - 1.6) | 3.0 | (1.1 - 4.9) | 0.656 |
| Parietal lobe | 1,090 | **-1.4** | **(-2.0 - -0.8)** | **2000.9** | 1.1 | (-0.3 - 2.5) | -3.5 | (-4.6 - -2.3) | **0.001** |
| Occipital lobe | 305 | **-1.5** | **(-2.4 - -0.6)** | 2000.6 | 0.6 | (-1.6 - 3.0) | -3.1 | (-5.0 - -1.3) | 0.351 |
| Others brain | 2,193 | **-2.0** | **(-2.5 - -1.5)** | 1988.9 | -7.6 | (-14.9 - 0.4) | -1.8 | (-2.4 - -1.3) | 1.000 |
| Contiguous sites | 2,460 | **0.6** | **(0.1 - 1.0)** | **2010.6** | 1.2 | (0.6 - 1.7) | -4.2 | (-7.3 - -0.9) | **0.042** |
| Brain unspecified | 963 | **-3.5** | **(-4.2 - -2.9)** | **2006.0** | -1.3 | (-2.4 - -0.2) | -9.9 | (-12.4 - -7.4) | **0.000** |
| **Histology** | | | | | | | | | |
| Global specified | 8,592 | **1.7** | **(1.4 - 2.0)** | **1997.1** | 4.5 | (3.2 - 5.8) | 0.7 | (0.2 - 1.2) | **0.000** |
| Gliomas and embryonal t. | 8,335 | **1.7** | **(1.4 - 2.0)** | **1997.6** | 4.2 | (3.0 - 5.5) | 0.7 | (0.2 - 1.3) | **0.000** |
| Glioblastoma | 3,859 | **3.8** | **(3.4 - 4.2)** | 2011.7 | 4.1 | (3.6 - 4.7) | 0.2 | (-2.9 - 3.4) | 0.219 |
| Meningiomas | 139 | -1.7 | (-3.1 - 0.2) | 1995.6 | 0.5 | (-6.5 - 8.0) | -2.1 | (-4.0 - -0.1) | 1.000 |
| Others | 118 | -0.7 | (-2.1 - 0.8) | 1987.0 | 999.9 | (-90.7 – 999.9) | -1.4 | (-3.1 - 0.4) | 0.222 |
| Unspecified | 2,624 | **-4.7** | **(-5.1 - -4.3)** | 1990.6 | -1.9 | (-5.5 - 1.8) | -5.0 | (-5.5 - -4.4) | 0.870 |

**APC**: Annual Percentage Change of the Adjusted Rate. **Join point**: Change point (JoinPoint) that determines Periods 1 and 2.

The p value indicates the level of statistical significance of the change in trend between the 2 periods. Bold indicates statistical significance (p<0.05)

**Table S2** Time trends in the incidence of central nervous system cancers in adult women. Spain, 1985-2015

|  |  | **1985-2015** | | **Join** | **Period 1** | | **Period 2** | | **P value** |
| --- | --- | --- | --- | --- | --- | --- | --- | --- | --- |
| **Tumoral type** | **N** | **APC** | **(95%CI)** | **point** | **APC** | **(95%CI)** | **APC** | **(95%CI)** |  |
| CNS | 9,108 | **0.6** | **(0.3 - 0.9)** | **1999.1** | 2.2 | (1.4 - 3.1) | -0.2 | (-0.7 - 0.3) | **0.001** |
| **Topography** | | | | | | | | | |
| Meninges | 204 | **-0.3** | **(-1.5 - 1.0)** | 2007.2 | 1.6 | (-0.3 - 3.6) | -6.2 | (-10.6 - -1.5) | 0.089 |
| Brain | 8,673 | **0.6** | **(0.3 - 0.8)** | **1999.7** | 1.9 | (1.1 - 2.7) | -0.2 | (-0.7 - 0.3) | **0.005** |
| Frontal lobe | 1,597 | **2.3** | **(1.8 - 2.9)** | 2007.6 | 1.9 | (1.0 - 2.8) | 3.5 | (1.5 - 5.6) | 1.000 |
| Temporal lobe | 1,295 | **2.0** | **(1.4 - 2.6)** | 2000.3 | 3.6 | (1.9 - 5.3) | 1.1 | (0.1 - 2.2) | 0.397 |
| Parietal lobe | 868 | **-0.8** | **(-1.4 - -0.1)** | **1994.2** | 7.7 | (3.7 - 1.8) | -2.0 | (-2.9 - -1.2) | **0.000** |
| Occipital lobe | 244 | -0.1 | (-1.1 - 0.9) | 2014.0 | 0.1 | (-1.0 - 1.3) | -10.8 | (-30.5 - 14.3) | 1.000 |
| Others brain | 1,781 | **-1.8** | **(-2.3 - -1.3)** | **2006.0** | -0.7 | (-1.5 - 0.2) | -4.6 | (-6.4 - -2.8) | **0.014** |
| Contiguous sites | 1,982 | **1.1** | **(0.6 - 1.6)** | 2013.8 | 1.0 | (0.4 - 1.5) | 5.9 | (-5.2 - 18.3) | 1.000 |
| Brain unspecified | 906 | **-1.5** | **(-2.2 - -0.8)** | **1996.3** | 4.6 | (2.0 - 7.3) | -3.5 | (-4.5 - -2.5) | **0.000** |
| **Histology** | | | | | | | | | |
| Global specified | 6,595 | **2,2** | **(1.9 - 2.6)** | **1999.8** | 4.7 | (3.7 - 5.8) | 0.9 | (0.2 - 1.5) | **0.000** |
| Gliomas and embryonal t. | 6,358 | **2.3** | **(1.9 - 2.6)** | **1998.8** | 4.6 | (3.5 - 5.7) | 1.3 | (0.8 - 1.9) | **0.000** |
| Glioblastoma | 2,864 | **4.1** | **(3.7 - 4.6)** | 2002.9 | 5.3 | (4.3 - 6.4) | 3.1 | (2.2 - 4.1) | 0.136 |
| Meningiomas | 144 | **0,5** | **(-1.0 - 2.0)** | 1991.7 | -4.7 | (-16.1 - 8.3) | 0.9 | (-0.9 - 2.6) | 1.000 |
| Others | 93 | **-2.2** | **(-3.8 - -0.4)** | 2007.3 | 0.1 | (-2.6 - 2.8) | -8.8 | (-14.8 - -2.4) | 0.269 |
| Unspecified | 2,513 | **-3.2** | **(-3.6 - -2.8)** | 1999.5 | -2.0 | (-3.0 - -1.0) | -4.1 | (-4.9 - -3.3) | 0.104 |

**APC**: Annual Percentage Change of the Adjusted Rate. **Join point**: Change point (JoinPoint) that determines Periods 1 and 2.

The p value indicates the level of statistical significance of the change in trend between the 2 periods. Bold indicates statistical significance (p<0.05)

**Table S3** Temporal trends in the incidence of malignant tumours of the central nervous system in boys. Spain, 1985-2015

|  |  | **1985-2015** | | **Join** | **Period 1** | | **Period 2** | | **P value** |
| --- | --- | --- | --- | --- | --- | --- | --- | --- | --- |
| **Tumoral type** | **N** | **APC** | **(95%CI)** | **point** | **APC** | **(95%CI)** | **APC** | **(95%CI)** |  |
| CNS | 1.275 | -0.3 | (-1.0 - 0.4) | **1991.3** | 8.4 | (2.1 - 15.1) | -1.1 | (-2.0 - -0.2) | **p= 0.043** |
| **Topography** | | | | | | | | | |
| Meninges | 7 | NOT EVALUABLE | | | | | | | |
| Brain | 1.123 | -0.4 | (-1.2 - 0.3) | 1987.0 | 39.5 | (8.7 - 79.0) | -1.3 | (-2.3 - -0.3) | p= 0.051 |
| Frontal lobe | 45 | NO EVALUABLE | | | | | | | |
| Temporal lobe | 60 | 0.2 | (-3.0 - 3.4) | 1996.4 | 6.0 | (-5.4 - 18.7) | -1.7 | (-6.3 - 3.2) | p= 1.000 |
| Parietal lobe | 32 | NOT EVALUABLE | | | | | | | |
| Occipital lobe | 11 | -0.2 | (-11.4 - 12.4) | 2000.1 | 3.7 | (-20.8 - 35.9) | -2.0 | (-16.5 - 15.1) | p= 1.000 |
| Others brain | 838 | -0.5 | (-1.4 - 0.4) | 1991.0 | 7.3 | (-1.1 - 16.4) | -1.1 | (-2.2 - 0.0) | p= 0.434 |
| Contiguous sites | 38 | -0.3 | (-4.3 - 3.8) | 1999.4 | 3.1 | (-6.3 - 13.4) | -3.0 | (-10.5 - 5.1) | p= 1.000 |
| Brain unspecified | 99 | -0.2 | (-2.5 - 2.2) | 1993.7 | 5.4 | (-5.4 - 17.4) | -1.5 | (-4.9 - 2.0) | p= 1.000 |
| **Histology** | | | | | | | | | |
| Global specified | 1.226 | -0.1 | (-0.9 - 0.6) | **1991.3** | 9.4 | (2.8 - 16.4) | -1.0 | (-1.9 - 0.0) | **p= 0.028** |
| Gliomas and embryonal t. | 1.143 | -0.4 | (-1.2 - 0.3) | **1991.4** | 9.0 | (2.5 - 16.0) | -1.3 | (-2.2 - -0.3) | **p= 0.028** |
| Glioblastoma | 32 | NOT EVALUABLE | | | | | | | |
| Meningiomas | 5 | NOT EVALUABLE | | | | | | | |
| Others | 78 | 0.3 | (-3.1 - 3.7) | 2010.6 | 2.3 | (-2.0 – 6.7) | -14.0 | (-29.4 - 4.9) | p= 0.709 |
| Unspecified | 49 | 0.0 | (-4.3 - 4.5) | 1988.0 | 15.6 | (-47.3 - 153.3) | -0.6 | (-6.0 - 5.0) | p= 1.000 |

**APC**: Annual Percentage Change of the Adjusted Rate. **Join point**: Change point (JoinPoint) that determines Periods 1 and 2.

The p value indicates the level of statistical significance of the change in trend between the 2 periods. Bold indicates statistical significance (p<0.05)

**Table S4** Temporal trends in the incidence of malignant tumours of the central nervous system in girls. Spain, 1985-2015

|  |  | **1985-2015** | | **Join** | **Period 1** | | **Period 2** | | **P value** |
| --- | --- | --- | --- | --- | --- | --- | --- | --- | --- |
| **Tumoral type** | **N** | **APC** | **(95%CI)** | **point** | **APC** | **(95%CI)** | **APC** | **(95%CI)** |  |
| CNS | 1097 | 0.2 | (-0.6 - 1.0) | 2011.0 | -0.5 | (-1.5 - 0.5) | 6.4 | (1.0 - 12.1) | p= 0.193 |
| **Topography** | | | | | | | | | |
| Meninges | 4 | 1.1 | (-22.4 - 31.7) | 1993.0 | 14.8 | (-88.8 - 999.9) | -0.1 | (-30.4 - 43.4) | p= 0.914 |
| Brain | 940 | 0.1 | (-0.7 - 0.9) | 1990.8 | 7.7 | (-0.2 - 16.2) | -0.5 | (-1.5 - 0.5) | p= 0.392 |
| Frontal lobe | 41 | 1.4 | (-3.2 - 6.2) | 2010.6 | 0.4 | (-5.4 - 6.6) | 7.0 | (-13.4 - 32.3) | p= 1.000 |
| Temporal lobe | 47 | NOT EVALUABLE | | | | | | | |
| Parietal lobe | 25 | NOT EVALUABLE | | | | | | | |
| Occipital lobe | 15 | NOT EVALUABLE | | | | | | | |
| Others brain | 718 | -0.3 | (-1.2 - 0.7) | 1991.5 | 6.2 | (-1.2 - 14.2) | -0.9 | (-2.0 - 0.3) | p= 0.570 |
| Contiguous sites | 25 | NOT EVALUABLE | | | | | | | |
| Brain unspecified | 69 | -0.5 | (-3.3 - 2.3) | 1997.9 | 2.7 | (-7.3 - 13.8) | -1.9 | (-6.8 - 3.3) | p= 1.000 |
| **Histology** |  |  |  |  |  |  |  |  |  |
| Global specified | 1038 | 0.3 | (-0.5 - 1.1) | 2011.0 | -0.4 | (-1.4 - 0.6) | 6.3 | (0.8 - 12.1) | p= 0.242 |
| Gliomas and embryonal t. | 975 | 0.1 | (-0.7 - 0.9) | 2011.2 | -0.5 | (-1.6 - 0.5) | 6.3 | (0.4 - 12.5) | p= 0.319 |
| Glioblastoma | 30 | 0.4 | (-5.1 - 6.3) | 2013.4 | -0.6 | (-6.9 - 6.3) | 22.0 | (-37.9 - 139.7) | p= 1.000 |
| Meningiomas | 4 | 1.1 | (-22.4 - 31.7) | 1993.0 | 14.8 | (-88.8 - 999.9) | -0.1 | (-30.4 - 43.4) | p= 0.914 |
| Others | 59 | 1.3 | (-2.7 - 5.5) | 1994.5 | 6.3 | (-19.0 - 39.5) | 0.5 | (-5.5 - 6.8) | p= 1.000 |
| Unspecified | 59 | 0.1 | (-3.2 - 3.5) | 1995.8 | 4.1 | (-6.8 - 16.4) | -1.2 | (-5.9 - 3.8) | p= 1.000 |

**APC**: Annual Percentage Change of the Adjusted Rate. **Join point**: Change point (JoinPoint) that determines Periods 1 and 2.

The p value indicates the level of statistical significance of the change in trend between the 2 periods. Bold indicates statistical significance (p<0.05)

**A**
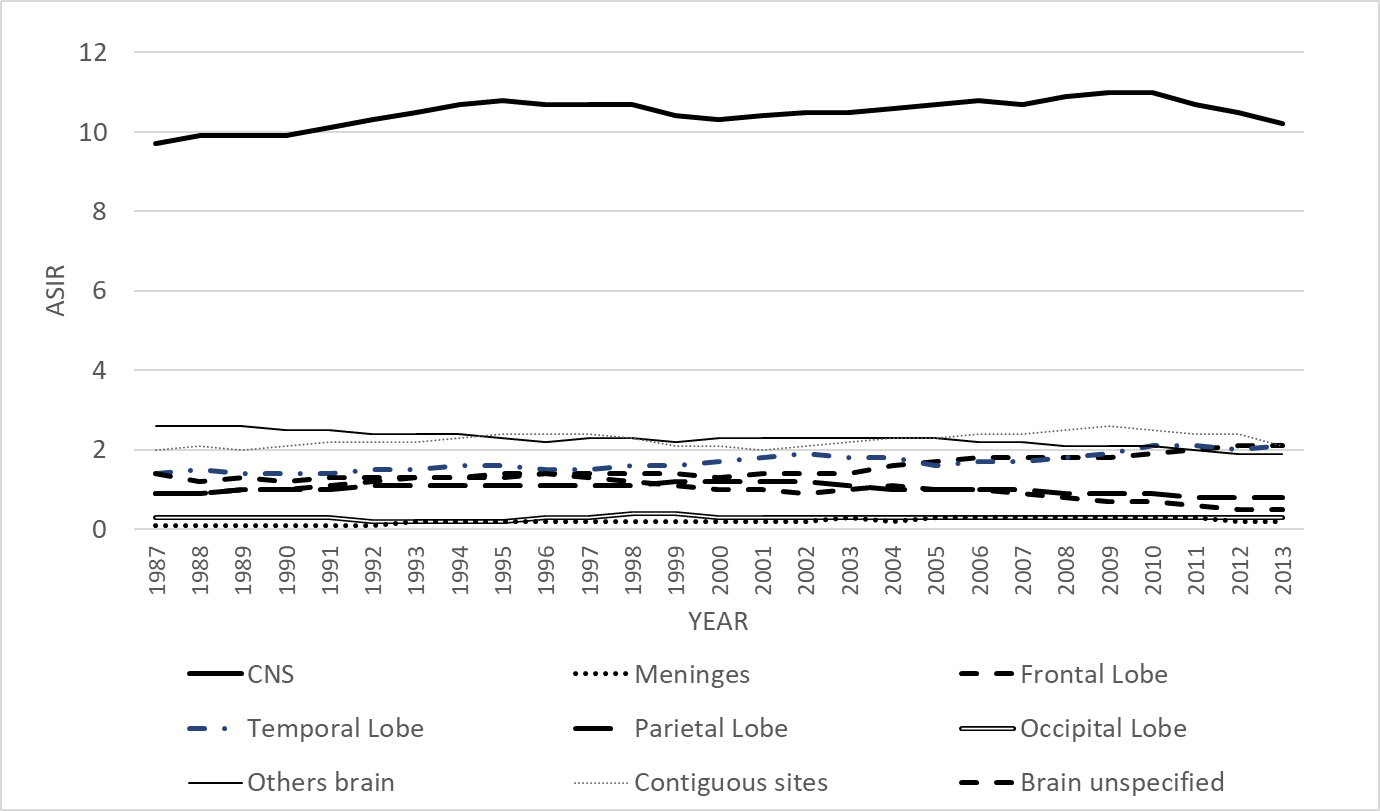


**B**
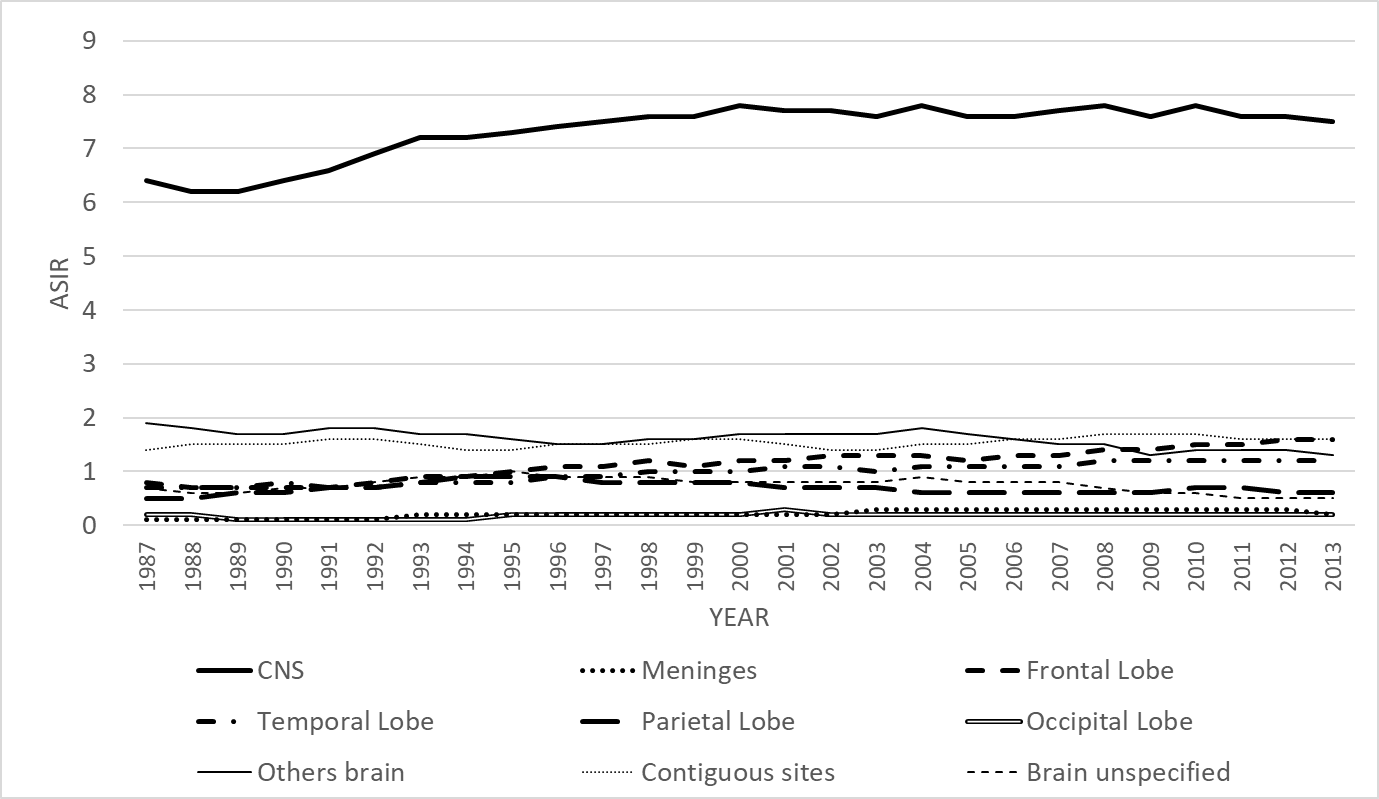


**Figure S1** (A) Evolution of incidence rates adjusted to the smoothed standard European population of malignant tumours of the central nervous system according to tumour subsite in adults. Spain. 1987-2013. Men. (B) Evolution of incidence rates adjusted to the smoothed standard European population of malignant tumours of the central nervous system according to tumour subsite in adults. Spain. 1987-2013. Women

**A**
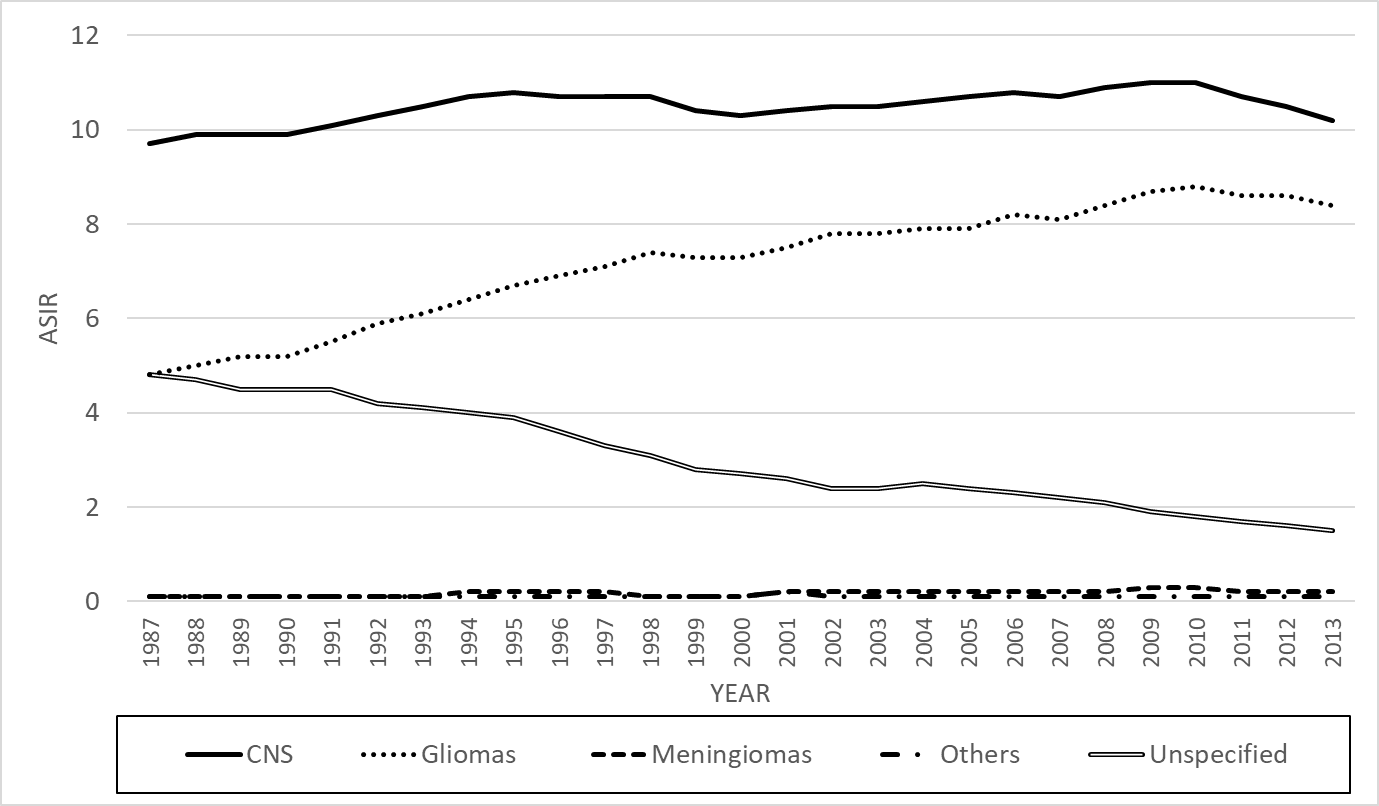


**B**
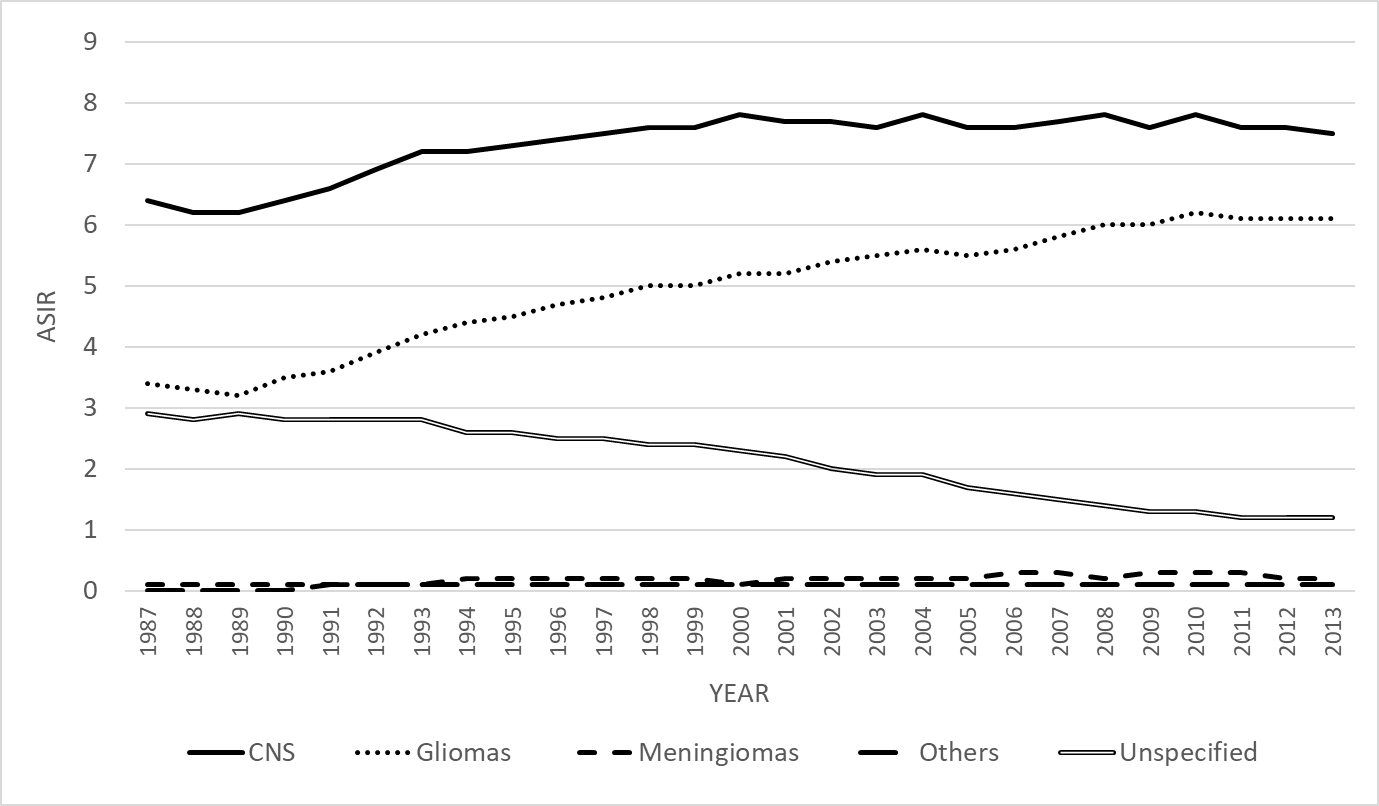


**Figure S2** (A) Evolution of incidence rates adjusted to the smoothed standard European population of malignant tumours of the central nervous system according to major histological groups. Spain, 1987-2013. Men. (B) Evolution of incidence rates adjusted to the smoothed standard European population of malignant tumours of the central nervous system according to major histological groups. Spain, 1987-2013. Women
